# The reversibility of cardiac damage after transcatheter aortic valve implantation and short-term outcomes in a real-world setting

**DOI:** 10.1093/ehjci/jeaf045

**Published:** 2025-02-04

**Authors:** Rinchyenkhand Myagmardorj, Federico Fortuni, Philippe Généreux, Takeru Nabeta, Jan Stassen, Xavier Galloo, Maria Chiara Meucci, Steele Butcher, Frank van der Kley, David J Cohen, Marie-Annick Clavel, Philippe Pibarot, Martin B Leon, Madelien V Regeer, Victoria Delgado, Nina Ajmone Marsan, Jeroen J Bax

**Affiliations:** Department of Cardiology, Heart Lung Center, Leiden University Medical Center, Leiden, The Netherlands; Department of Cardiology, Mongolian National University of Medical Sciences, Ulaanbaatar, Mongolia; Department of Cardiology, Heart Lung Center, Leiden University Medical Center, Leiden, The Netherlands; Cardiology and Cardiovascular Pathophysiology, S. Maria della Misericordia Hospital, University of Perugia, Perugia, Italy; Gagnon Cardiovascular Institute, Morristown Medical Center, Morristown, NJ, USA; Department of Cardiology, Heart Lung Center, Leiden University Medical Center, Leiden, The Netherlands; Department of Cardiology, Heart Lung Center, Leiden University Medical Center, Leiden, The Netherlands; Department of Cardiology, Jessa Hospital, Hasselt, Belgium; Department of Cardiology, Heart Lung Center, Leiden University Medical Center, Leiden, The Netherlands; Department of Cardiology, UZ Brussel, Vrije Universiteit Brussel, Brussels, Belgium; Department of Cardiology, Heart Lung Center, Leiden University Medical Center, Leiden, The Netherlands; Department of Cardiology, Heart Lung Center, Leiden University Medical Center, Leiden, The Netherlands; Department of Cardiology, Heart Lung Center, Leiden University Medical Center, Leiden, The Netherlands; Cardiovascular Research Foundation, New York, NY, USA; St. Francis Hospital and Heart Center, Roslyn, NY, USA; Department of Cardiology, Québec Heart and Lung Institute - Laval University, Québec, Canada; Department of Cardiology, Québec Heart and Lung Institute - Laval University, Québec, Canada; Cardiovascular Research Foundation, New York, NY, USA; Division of Cardiology, NewYork-Presbyterian Hospital/Columbia University Irving Medical Center, New York, NY, USA; Department of Cardiology, Heart Lung Center, Leiden University Medical Center, Leiden, The Netherlands; Department of Cardiovascular Imaging, Hospital University Germans Trias i Pujol, Barcelona, Spain; Department of Cardiology, Heart Lung Center, Leiden University Medical Center, Leiden, The Netherlands; Department of Cardiology, Heart Lung Center, Leiden University Medical Center, Leiden, The Netherlands; Department of Cardiology, Turku Heart Center, University of Turku and Turku University Hospital, Turku, Finland

**Keywords:** aortic stenosis, echocardiography, prognosis, transcatheter aortic valve implantation

## Abstract

**Aims:**

This study aims to assess the changes in cardiac damage stage in a real-world cohort of patients undergoing transcatheter aortic valve implantation (TAVI), and to investigate the prognostic value of cardiac damage stage evolution.

**Methods and results:**

Patients with severe aortic stenosis (AS) undergoing TAVI were retrospectively analysed. A five-stage system based on the presence and extent of cardiac damage assessed by echocardiography was applied before and 6 months after TAVI. Multivariable Cox regression analyses were used to examine independent prognostic value of the changes in cardiac damage after TAVI. A total of 734 patients with severe AS (mean age, 79.8 ± 7.4 years; 55% male) were included. Before TAVI, 32 (4%) patients did not show any sign of extra-valvular cardiac damage (Stage 0), 85 (12%) had left ventricular damage (Stage 1), 220 (30%) left atrial and/or mitral valve damage (Stage 2), 227 (31%) pulmonary vasculature and/or tricuspid valve damage (Stage 3), and 170 (23%) right ventricular damage (Stage 4). Six months after TAVI, 39% of the patients improved at least one stage in cardiac damage. Staging of cardiac damage at 6 months after TAVI [hazard ratio (HR) per one-stage increase, 1.391; *P* = 0.035] as well as worsening in the stage of cardiac damage (HR, 3.729; *P* = 0.005) were independently associated with 2-year all-cause mortality.

**Conclusion:**

More than one-third of patients with severe AS showed an improvement in cardiac damage 6 months after TAVI. Staging cardiac damage at baseline and follow-up may improve risk stratification in patients undergoing TAVI.

## Graphical Abstract

**Graphical Abstract:**
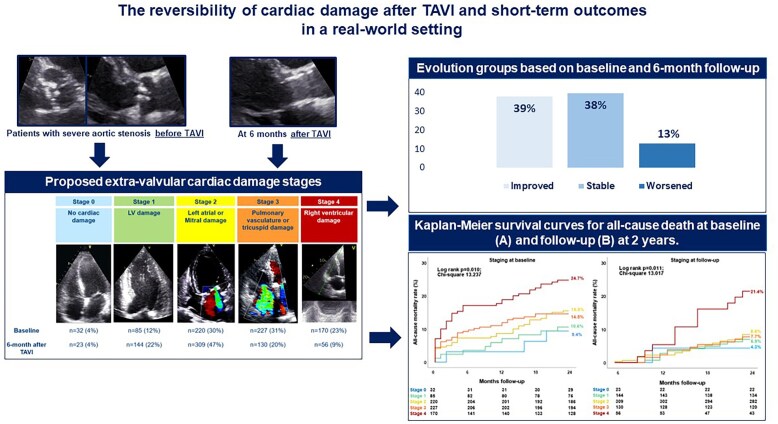

## Introduction

Calcific aortic valve disease is the most common valvular heart disease requiring valve replacement in the aging population of the Western world.^1^ Transcatheter aortic valve implantation (TAVI) or surgical aortic valve replacement (AVR) are recommended in patients with symptomatic severe aortic stenosis (AS) or with reduced left ventricular systolic function due to AS-related cardiac remodelling.^2–4^ If left untreated, not only does the severity of AS worsen but also progressive extra-valvular cardiac damage occurs, extending from the aortic valve to the left ventricle (LV, left atrium (LA), pulmonary circulation, and eventually the right ventricle (RV) and tricuspid valve.^5^ In 2017, Généreux *et al*.^6^ proposed a staging system for AS-related extra-valvular cardiac damage and created models for prognostication of patients with severe AS, based on the extent of extra-valvular cardiac damage. Subsequent studies applied this ‘staging concept’ in patients with severe AS^7–9^ and other valvular heart diseases.^10,11^ More recently, Généreux *et al*.^6^ applied the extra-valvular cardiac damage staging system to the PARTNER II and III (Placement of AoRTic TraNscathetER Valves) patient cohorts for risk stratification^12^ considering both surgical and transcatheter AVR in the context of the highly selected population included in these randomized controlled trials. The authors demonstrated that the cardiac damage staging system applied before and 1 year after AVR was predictive of patient outcomes.^12^ However, the changes in cardiac damage and its relative prognostic value specifically after TAVI in a real-world setting have not been investigated. Accordingly, the present study aims to (i) evaluate the change in the staging of cardiac damage 6 months after TAVI using the criteria introduced by Généreux *et al*.^6^ and (ii) to assess the prognostic value (all-cause mortality) of the change in cardiac damage after TAVI in a real-world cohort of patients with severe AS.

## Methods

### Patient population and data collection

**Figure 1:**
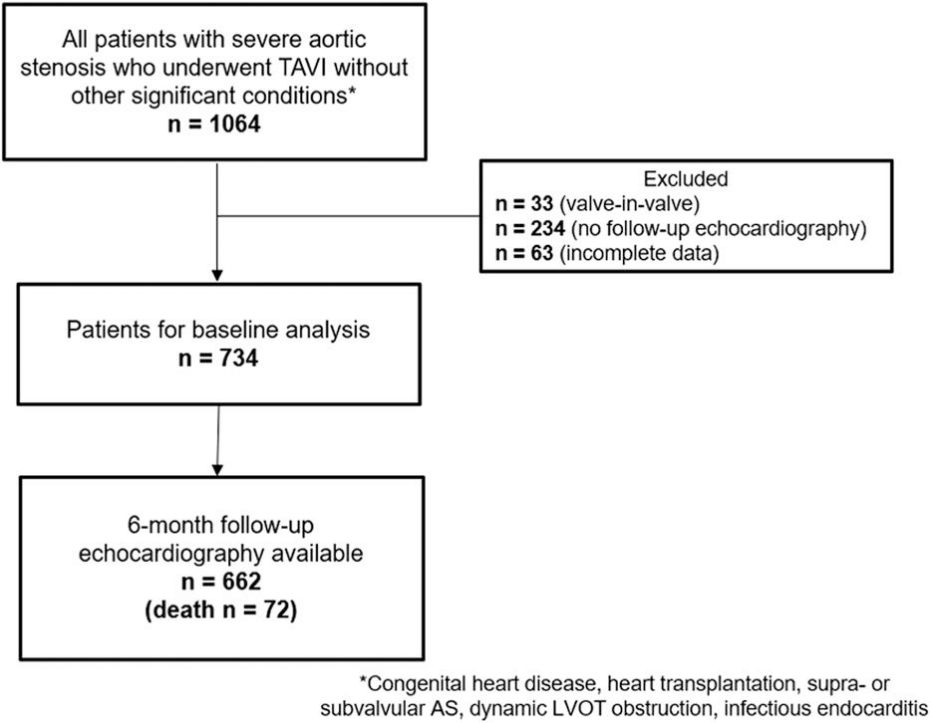
Patient inclusion flow-chart. AS, aortic stenosis; FU, follow-up; LVOT, left ventricular outflow tract; TAVI, transcatheter aortic valve implantation.

Patients with severe AS who underwent TAVI between November 2007 and December 2019 at the Leiden University Medical Center (The Netherlands) were included. Severe AS was defined as an aortic valve area assessed with the continuity equation <1.0 cm^2^ (or an indexed aortic valve area <0.6 cm^2^/m^2^) and/or a mean aortic valve gradient ≥40 mmHg and/or a peak aortic jet velocity ≥4 m/s, according to current echocardiographic guidelines.^13^ Moreover, patients with low-flow low-gradient AS were also included (defined by aortic valve area <1.0 cm^2^, mean aortic transvalvular pressure gradient <40 mmHg, LV ejection fraction <50%, and stroke volume index <35 mL/m^2^).^13^ The exclusion criteria were congenital heart disease, cardiac transplantation, supra- or subvalvular AS, dynamic LV outflow tract obstruction, infective endocarditis, and valve-in-valve procedures. Patients with incomplete baseline or follow-up echocardiographic data or those who died within 6-month follow-up were excluded (*Figure 1*). All patients underwent complete clinical evaluation before TAVI. Patient information was retrospectively collected from electronic medical records of the Leiden University Medical Centre (EPD-Vision 11.8.4.0) and hospital records (HiX; ChipSoft, Amsterdam, The Netherlands). Clinical data included demographic characteristics, symptoms, comorbidities, laboratory tests, and medication. The date when the TAVI was performed was recorded and considered as a dichotomous variable (before vs. after 2015) for adjustment in the analysis of prognosis. Since this is a retrospective analysis of clinically collected data, the Institutional Review Board approved the study and waived the need for patient written informed consent.

### Transthoracic echocardiography

All patients underwent echocardiographic evaluation before and 6 months after TAVI. Echocardiographic data were acquired using available ultrasound systems (Vivid-7, E9 and E95; GE Healthcare, Horten, Norway). The ultrasound systems were equipped with M3S, M5S, M5Sc-D, and 4Vc-D 4D matrix cardiac probes. Two-dimensional, colour, continuous- and pulsed-wave Doppler images were obtained from the parasternal, apical, and subcostal views. All images were digitally stored for offline analysis (EchoPAC version 203; GE-Vingmed, Horten, Norway). From the parasternal long-axis view, LV dimensions were assessed, and LV mass was calculated using Devereux’s formula and indexed to body surface area (LV mass index).^14^ LV end-diastolic and end-systolic volumes were evaluated using the apical 2- and 4-chamber views, and LV ejection fraction was calculated according to the biplane Simpson’s method. Using the biplane method of disks, LA volumes were measured at end-systole in the apical 2- and 4-chamber views and subsequently indexed to body surface area (LA volume index).^14^ Pulsed-wave Doppler recordings of the transmitral flow were used to measure peak early (E) and late (A) diastolic velocities for the assessment of LV diastolic function.^15^ Using tissue Doppler imaging of the mitral annulus on the apical 4-chamber view, the e’ was obtained at both the lateral and septal side of the mitral annulus, and the values were averaged to calculate the E/e’ ratio for estimation of LV filling pressures.^15^ Grading of mitral and tricuspid regurgitation was based on current recommendations.^16^ The pulmonary artery systolic pressure (PASP) was calculated from the peak velocity of the tricuspid regurgitant jet using the Bernoulli equation, adding the right atrial pressure determined by the inspiratory collapse and diameter of the inferior vena cava.^17^ To evaluate RV systolic function, anatomical M-mode was applied on the focused apical 4-chamber view of the RV to measure tricuspid annular plane systolic excursion (TAPSE).^17^

Aortic valve area calculation was performed by the continuity equation using velocity time integrals of the aorta and LV outflow tract. Peak and mean aortic transvalvular gradients were calculated using the modified Bernoulli equation.^13^ Continuous and pulsed wave Doppler data were obtained from the apical 5- or 3-chamber views.

### Definition of modified extra-valvular cardiac damage classification

**Figure 2:**
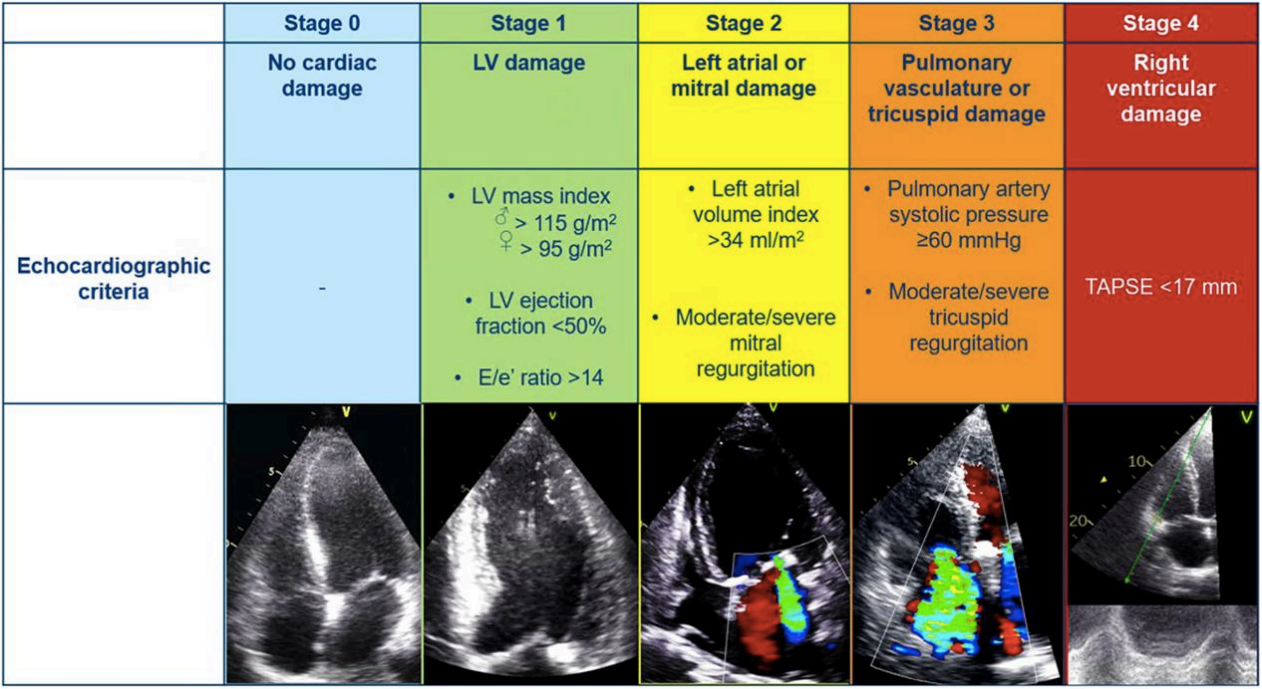
Stages of cardiac damage in severe aortic stenosis. The Figure illustrates the echocardiographic-based cardiac damage staging system that was applied before and after TAVI. LV, left ventricular; TAPSE, tricuspid annular plane systolic excursion; TAVI, transcatheter aortic valve implantation.

Patients were classified into five independent stages based on the presence and extent of cardiac damage, as proposed by Généreux *et al*.^6^ This classification included the following: Stage 0 = no signs of cardiac damage; Stage 1 = LV damage, defined as LV ejection fraction <50% and/or *E*/*e*´>14 and/or LV mass index >115 g/m^2^ (male) or > 95 g/m^2^ (female); Stage 2 = LA or mitral valve damage, defined as ≥ moderate mitral regurgitation and/or indexed LA volume >34 mL/m^2^; Stage 3 = pulmonary or tricuspid valve damage, defined as PASP ≥60 mmHg and/or ≥ moderate tricuspid regurgitation; Stage 4 = RV damage, defined as TAPSE <17 mm^2^ (see *Figure 2*). Stage 3 was also divided into 2 groups based on PASP values <60 mmHg (Stage 3a) or ≥60 mmHg (Stage 3b) as proposed by Okuno *et al*.^18^ Patients were hierarchically classified as a given stage (the most severe stage) if at least one of the proposed criteria was met within that stage. In contrast to a previous study,^12^ atrial fibrillation was not included in this staging classification because reversibility of atrial fibrillation is unlikely after TAVI. Cardiac damage was evaluated before and 6 months after TAVI. An improvement in the stage of cardiac damage was defined as an improvement of at least 1 stage at follow-up, whereas worsening in cardiac damage staging was defined as worsening of at least 1 stage at follow-up. Stabilization of cardiac damage was considered if baseline and follow-up staging category were similar.

### Clinical endpoints and follow-up

Patients were followed up for all-cause mortality after TAVI at 2 years. The median time interval between TAVI and the follow-up echocardiographic assessment was 6 months, and 87% of the follow-up assessments were performed within 5–7 months after TAVI. Survival data were complete for all patients and collected from the departmental cardiology information system, which is linked to the municipal civil registries.

### Statistical analysis

Categorical variables are presented as counts and percentages and compared using the χ^2^ test. Normally distributed continuous variables are expressed as mean ± standard deviation, while non-normally distributed continuous variables are presented as median and interquartile range. Continuous data were compared with one-way ANOVA applying the Bonferroni correction for normally distributed variables, whereas the Kruskal–Wallis test was used for variables with a non-normal distribution. Survival analysis was performed with the Kaplan–Meier method. To compare proportion differences between stages of cardiac damage at baseline and follow-up, McNemar test was used. Cumulative event rates were compared across groups using the log-rank test. For evaluating the association between the staging classification (as well as other clinical and echocardiographic parameters) and all-cause mortality, multivariable Cox regression analyses were performed after identification of clinically significant variables at the univariable Cox regression analyses. A landmark analysis was performed to evaluate the association between the stage of cardiac damage at 6 months and the 2-year outcomes. Moreover, the change in cardiac damage staging from baseline to 6-month follow-up was examined, and a multivariable Cox regression analysis was performed to investigate the association between this change and all-cause mortality at 2-year follow-up.^19^ A two-sided *P*-value <0.05 was considered statistically significant. Statistical analysis was performed using IBM SPSS version 25.0 (SPSS Inc., IBM Corp).

## Results

### Baseline characteristics

A total of 734 patients with severe AS (mean age 79.8 ± 7.4 years, 55% male) were included and categorized into 5 groups, based on the modified AS staging system. Thirty-two (4%) patients did not show any sign of extra-valvular cardiac damage (Stage 0), 85 (12%) patients had LV damage (Stage 1), 220 (30%) patients had LA and/or mitral valve damage (Stage 2), 227 (31%) patients had pulmonary vasculature and/or tricuspid valve damage (Stage 3), and 170 (23%) patients had RV damage (Stage 4). The prevalence of atrial fibrillation, previous cardiac surgery, higher risk score [European System for Cardiac Operative Risk Evaluation (EuroSCORE) II] increased in parallel to more severe cardiac damage; also impaired renal function, use of diuretics, and higher heart rate were associated with more advanced stages of cardiac damage (*Table 1*).

**Table 1:** Baseline clinical characteristics according to cardiac damage staging

	Total population (*n* = 734)	Baseline					*P*-value
		Stage 0 (*n* = 32)	Stage 1 (*n* = 85)	Stage 2 (*n* = 220)	Stage 3 (*n* = 227)	Stage 4 (*n* = 170)	
Age (years)	79.8 ± 7.4	78.7 ± 7.8	78.0 ± 8.4	80.0 ± 5.9	81.0 ± 7.0 ^a^	79.0 ± 8.6	**0**.**008**
Male gender, *n* (%)	400 (55)	17 (53)	53 (62)	121 (55)	107 (47)	102 (60)	0.056
Body mass index (kg/m^2^)	26.6 ± 4.5	26.4 ± 3.9	27.2 ± 6.2	27.3 ± 4.4	25.7 ± 4.2 ^b^	26.4 ± 3.7	**0**.**001**
Body surface area (m^2^)	1.9 ± 0.2	1.9 ± 0.2	1.9 ± 0.2	1.9 ± 0.2	1.8 ± 0.2^b^	1.9 ± 0.2	**0**.**006**
Hypertension, *n* (%)	548 (76)	21 (68)	68 (81)	166 (77)	167 (75)	126 (75)	0.593
Diabetes mellitus, *n* (%)	211 (29)	6 (19)	29 (35)	74 (34.3)	48 (22)	54 (32.0)	**0**.**016**
Atrial fibrillation, *n* (%)	138 (19)	1 (3)	3 (4)	25 (11)	45 (20)	64 (38)	**<0**.**0001**
Pacemaker, *n* (%)	89 (12)	3 (9)	5 (6)	21 (10)	32 (14)	28 (17)	0.072
Dyslipidaemia, *n* (%)	469 (65)	21 (68)	48 (57)	141 (65)	149 (67)	110 (65)	0.609
Coronary artery disease, *n* (%)	440 (60)	15 (48)	51 (60)	126 (58)	130 (58)	118 (69)	0.067
Cardiac surgery, *n* (%)	150 (20)	3 (9)	13 (15)	40 (18)	32 (14)	62 (37)	**<0**.**0001**
Myocardial infarction, *n* (%)	162 (22)	4 (13)	21 (25)	38 (18)	50 (22)	49 (29)	0.063
Smoking, *n* (%)	148 (21)	5 (18)	26 (31)	47 (23)	47 (23)	35 (22)	0.081
Chronic obstructive pulmonary disease, *n* (%)	142 (22)	10 (33)	18 (26)	45 (23)	33 (18)	36 (23)	0.318
Peripheral artery disease, *n* (%)	207 (29)	10 (32)	26 (31)	61 (28)	54 (24)	56 (33)	0.374
EuroSCORE II, *n* (%)	3.3 (2.1–5.3)	1.9 (1.5–3.5)	2.7 (1.7–4.4)	3.0 (1.9–4.8)	3.0 (2.1–4.6)	4.6 (2.8–8.4)^a,b,c,d^	**<0**.**0001**
NYHA class III or IV, *n* (%)	415 (58.4)	16 (53)	40 (49)	115 (54)	136 (62)	108 (65)	0.064
Haemoglobin (g/dL)	12.5 ± 1.7	13.1 ± 1.5	12.8 ± 1.6	12.4 ± 1.9	12.3 ± 1.7	12.6 ± 1.6	**0**.**025**
Creatinine (mg/dL)	1.0 (0.8–1.3)	1.0 (0.9–1.2)	1.0 (0.8–1.4)	1.0 (0.9–1.3)	1.0 (0.8–1.2)	1.1 (0.9–1.5)	**<0**.**0001**
Heart rate (beats per minute)	71 ± 13	70 ± 11	69 ± 10	68 ± 12	71 ± 14	76 ± 15^a,b,d^	**<0**.**0001**
Systolic blood pressure (mmHg)	138 ± 23	142 ± 21	141 ± 22	141 ± 23	138 ± 23	132 ± 23 ^a,b^	**0**.**001**
Diastolic blood pressure (mmHg)	68 ± 13	69 ± 10	69 ± 13	67 ± 12	69 ± 14	69 ± 12	0.439
Medication							
Beta-blocker, *n* (%)	433 (61)	17 (55)	49 (59)	122 (57)	130 (59)	115 (69)	0.145
ACEi/ARB, *n* (%)	394 (55)	19 (61)	44 (53)	119 (55)	112 (51)	100 (60)	0.443
Calcium antagonist, *n* (%)	186 (26)	6 (19)	22 (27)	61 (28)	46 (21)	51 (31)	0.191
Diuretics, *n* (%)	407 (57)	16 (52)	36 (43)	113 (53)	130 (59)	112 (67)	**0**.**004**
Aspirin, *n* (%)	335 (48)	20 (67)	48 (59)	112 (53)	94 (43)	61 (37)	**0**.**001**
OAC/NOAC, *n* (%)	273 (39)	4 (13)	20 (25)	56 (26)	96 (44)	97 (59)	**<0**.**0001**
Statin, *n* (%)	468 (65)	21 (68)	49 (59)	149 (69)	141 (64)	108 (65)	0.523

Continuous variables are presented as mean ± SD or median (interquartile range).

Categorical variables are expressed as number (percentage).

*P*-values depict differences between stages of cardiac damage and are calculated by ANOVA and Kruskal–Wallis *H* test for continuous data (with normal and non-normal distribution, respectively), and by χ^2^ test for categorical data. Bold values represent significant *P*-values (< 0.05).

^a^ *P*-value <0.05 vs. Stage 1 with Bonferroni *post hoc* analysis.

^b^ *P*-value <0.05 vs. Stage 2 with Bonferroni *post hoc* analysis.

^c^ *P*-value <0.05 vs. Stage 0 with Bonferroni *post hoc* analysis.

^d^ *P*-value <0.05 vs. Stage 3 with Bonferroni *post hoc* analysis.

ACEi, angiotensin-converting enzyme inhibitor; ARB, angiotensin II receptor blocker; EuroSCORE, European system for cardiac operative risk evaluation; NOAC, non-vitamin K oral anticoagulant; NYHA, New York Heart Association; OAC, oral anticoagulant.

Regarding echocardiographic parameters, patients with advanced stages of cardiac damage had significantly larger LV and LA volumes, worse LV systolic and diastolic function, higher prevalence of significant mitral and tricuspid regurgitation, increased PASP, and worse RV systolic function compared with less advanced stages (*Table 2*).

**Table 2:** Baseline echocardiographic characteristics according to cardiac damage staging

	Total population (*n* = 734)	Baseline					*P*-value
		Stage 0 (*n* = 32)	Stage 1 (*n* = 85)	Stage 2 (*n* = 220)	Stage 3 (*n* = 227)	Stage 4 (*n* = 170)	
Valve morphology							0.816
Tricuspid	658 (96)	29 (94)	77 (98)	195 (96)	203 (97)	154 (95)	
Bicuspid	27 (4)	2 (6)	2 (2)	8 (4)	7 (3)	8 (5)	
LV end-diastolic diameter indexed (mm/m^2^)	25.2 ± 4.5	21.9 ± 3.6	25.4 ± 4.0**^a^**	25.0 ± 4.1**^a^**	25.3 ± 4.5**^a^**	25.9 ± 5.1**^a^**	**<0.0001**
LV end-systolic diameter indexed (mm/m^2^)	18.2 ± 5.3	15.3 ± 4.1	18.3 ± 4.6	17.7 ± 5.1	18.1 ± 5.2	19.4 ± 6.1**^a,b^**	**<0**.**0001**
Septal wall thickness (mm)	13.3 ± 2.8	11.8 ± 2.2	13.1 ± 2.5	13.5 ± 2.9**^a^**	13.6 ± 2.8**^a^**	13.0 ± 2.7	**0**.**004**
Posterior wall thickness (mm)	12.3 ± 2.3	10.9 ± 2.0	11.9 ± 1.9	12.8 ± 2.5**^a,c,d^**	12.1 ± 2.1**^a^**	12.4 ± 2.4**^a^**	**<0**.**0001**
Relative wall thickness (%)	0.6 ± 0.2	0.6 ± 0.2	0.5 ± 0.1	0.6 ± 0.2	0.6 ± 0.2	0.5 ± 0.2	0.246
LV end-diastolic volume (mL/m^2^)	47.9 (37.6–64.1)	37.1 (29.7–43.3)	45.0 (35.9–56.2)	48.2 (37.9–63.0)**^a^**	49.2 (38.7–64.3)**^a^**	51.2 (37.5–74.1)**^a,c^**	**<0**.**0001**
LV end-systolic volume (mL/m^2^)	20.1 (13.3–32.2)	12.0 (8.8–15.3)	18.4 (12.1–26.1)	19.8 (13.6–30.7)**^a^**	20.5 (13.7–31.9)**^a^**	26.1 (15.1–46.2)**^a,b,c,d^**	**<0**.**0001**
LV mass index (g/m^2^)	126.3 ± 38.7	83.6 ± 16.8	124.1 ± 34.9**^a^**	130.1 ± 38.3**^a^**	127.2 ± 39.5**^a^**	128.9 ± 38.4**^a^**	**<0**.**0001**
LV ejection fraction (%)	58.0 (46.0–65.0)	66.0 (60.5–71.0)	60.1 (50.2–66.5)**^a^**	60 (52.0–66.3)**^a^**	58.0 (47.0–65.0)**^a^**	48.0 (37.0–60.0)**^a,b,c,d^**	**<0**.**0001**
E/e’ ratio	16.6 (12.0–24.2)	9.9 (8.5–12.0)	15.8 (11.5–20.8)**^a^**	17.0 (12.5–24.9)**^a^**	16.8 (12.4–25.0)**^a,c^**	17.5 (12.8–25.6)**^a^**	**<0**.**0001**
Left atrial volume index (mL/m^2^)	44.4 ± 16.5	24.7 ± 4.5	27.3 ± 4.5	47.0 ± 11.4**^a,c^**	47.9 ± 18.4**^a,c^**	49.2 ± 16.3**^a,c^**	**<0**.**0001**
Significant mitral regurgitation, *n* (%)	155 (22)	—	—	37 (17)	68 (31)	50 (30)	**<0**.**0001**
Systolic pulmonary artery pressure (mmHg)	34.1 ± 15.1	26.7 ± 11.3	26.2 ± 14.2	28.8 ± 13.4	40.7 ± 13.4**^a,b,c^**	37.6 ± 15.7**^a,b,c^**	**<0**.**0001**
Significant tricuspid regurgitation, *n* (%)	322 (44)	—	—	—	220 (97)	102 (60)	**<0**.**0001**
Tricuspid annular plane systolic excursion (mm)	18.7 ± 4.5	19.6 ± 2.5	20.5 ± 4.0	21.0 ± 3.6**^c^**	20.1 ± 3.1	13.1 ± 2.0**^a,b,c,d^**	**<0**.**0001**
Stroke volume index (mL/m^2^)	39.0 ± 12.5	40.3 ± 10.9	39.3 ± 12.0	41.3 ± 12.9	40.6 ± 12.9	33.4 ± 10.2**^a,b,c,d^**	**<0**.**0001**
Mean aortic valve gradient (mmHg)	41.1 ± 17.4	44.1 ± 14.3	43.8 ± 18.6	45.3 ± 17.8	40.9 ± 17.0	34.3 ± 15.1**^a,b,c,d^**	**<0**.**0001**
Peak aortic jet velocity (m/s)	3.9 ± 0.8	4.1 ± 0.6	4.0 ± 0.8	4.1 ± 0.8	3.9 ± 0.8**^b^**	3.6 ± 0.8**^a,b,c,d^**	**<0**.**0001**
Indexed aortic valve area (cm^2^/m^2^)	0.4 ± 0.2	0.4 ± 0.1	0.5 ± 0.2	0.4 ± 0.2	0.5 ± 0.2	0.4 ± 0.2	0.623

Continuous variables are presented as mean ± SD or median (interquartile range). Categorical variables are expressed as number (percentage).

*P*-values depict differences between stages of cardiac damage and are calculated by ANOVA and Kruskal–Wallis *H* test for continuous data (with normal and non-normal distribution, respectively), and by χ2 test for categorical data. Bold values represent significant *P*-values (< 0.05).

^a^ *P*-value <0.05 vs. Stage 0 with Bonferroni *post hoc* analysis.

^b^ *P*-value <0.05 vs. Stage 2 with Bonferroni *post hoc* analysis.

^c^ *P*-value <0.05 vs. Stage 1 with Bonferroni *post hoc* analysis.

^d^ *P*-value <0.05 vs. Stage 3 with Bonferroni *post hoc* analysis.

AS, aortic stenosis; LV, left ventricular.

### Association between cardiac damage staging and 2-year all-cause mortality

**Figure 3:**
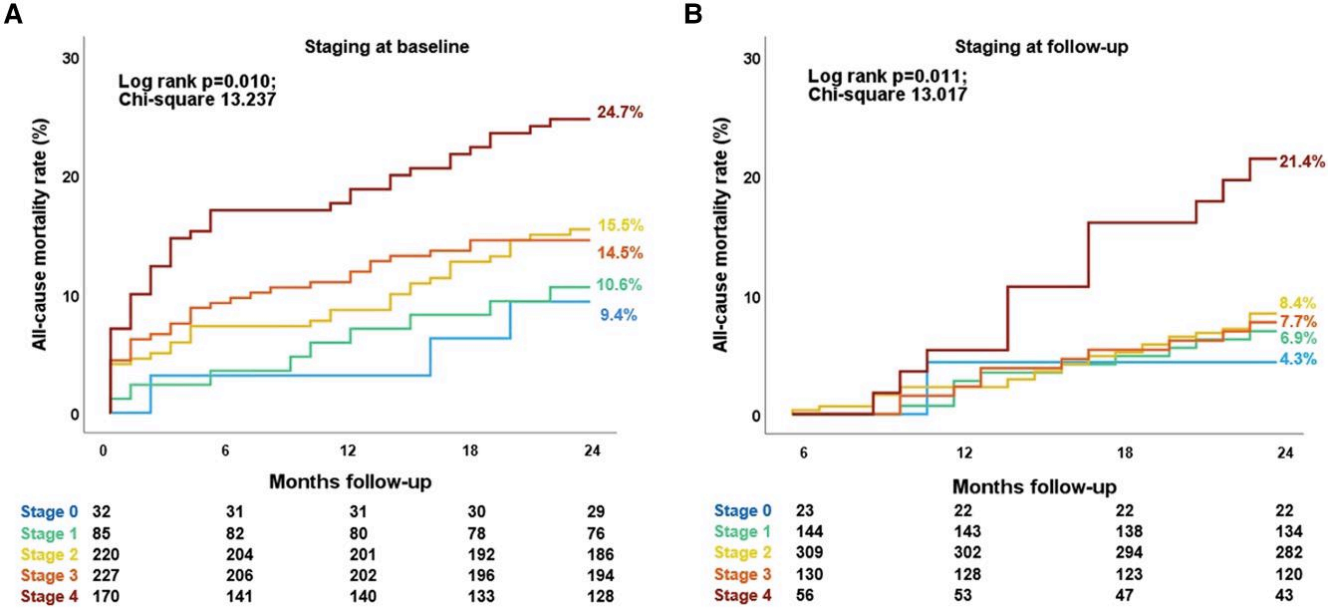
Kaplan–Meier survival curves for all-cause death according to cardiac damage assessed at baseline (A) and 6-month follow-up (B). Both in the baseline (A) and follow-up (B) survival analysis, cardiac damage stages from 0 to 3 had significantly lower event rates compared to Stage 4 based on pairwise comparison analysis (*P*-value <0.05 for all pairwise comparisons with all the other cardiac damage stages).

At 2-year follow-up, 121 patients (17%) had died. Based on the cardiac damage staging performed at baseline (before TAVI), the estimated 2-year mortality rate was 9.4% for patients who were in Stage 0, 10.6% in Stage 1, 15.5% in Stage 2, 14.5% in Stage 3, and 24.7% in Stage 4 (overall log-rank *P* = 0.010; *Figure 3A*). Interestingly, when Stage 3 was subdivided according to PASP values, as proposed by Okuno *et al*.,^18^ the 2-year survival rate of patients on Stage 3b (PASP ≥ 60 mmHg) was similar to those on Stage 4 whereas the one of those on Stage 3a was similar to the patients on Stage 2 (see Supplementary data online, *Figure S1*). Focusing on cardiac damage staging 6 months after TAVI, the estimated 2-year mortality rate based on this landmark analysis was the highest in Stage 4 (21.4%), as compared with Stage 0 (4.3%), Stage 1 (6.9%), Stage 2 (8.4%), and Stage 3 (7.7%; overall log-rank *P* = 0.011; *P-*value <0.05 for all pairwise comparisons between Stage 4 and all the other stages; *Figure 3B*), and this was consistent also after stratifying Stage 3 according to PASP values into 3a and 3b (see Supplementary data online, *Figure S1*).

Supplementary data online, *Table S1* summarizes the uni- and multivariable Cox regression analysis for all-cause mortality to assess the prognostic value of the cardiac damage staging system at baseline. On multivariable analysis, apart from baseline cardiac damage [hazard ratio (HR) per 1 stage increase, 1.341; 95% confidence interval (CI), 1.098–1.637; *P* = 0.004], also chronic obstructive pulmonary disease (HR, 2.221; 95% CI, 1.457–3.386; *P* < 0.001), serum haemoglobin level (HR, 0.857; 95% CI, 0.772–0.951; *P* = 0.004), creatinine level (HR, 1.172; 95% CI, 1.003–1.370; *P* = 0.046), and earlier TAVI implantation (performed before year 2015) compared with more recent implantation (after 2015) were independently associated with 2-year all-cause mortality (see Supplementary data online, *Table S1*).

### Evolution of cardiac damage and relation with 2-year outcomes

**Figure 4:**
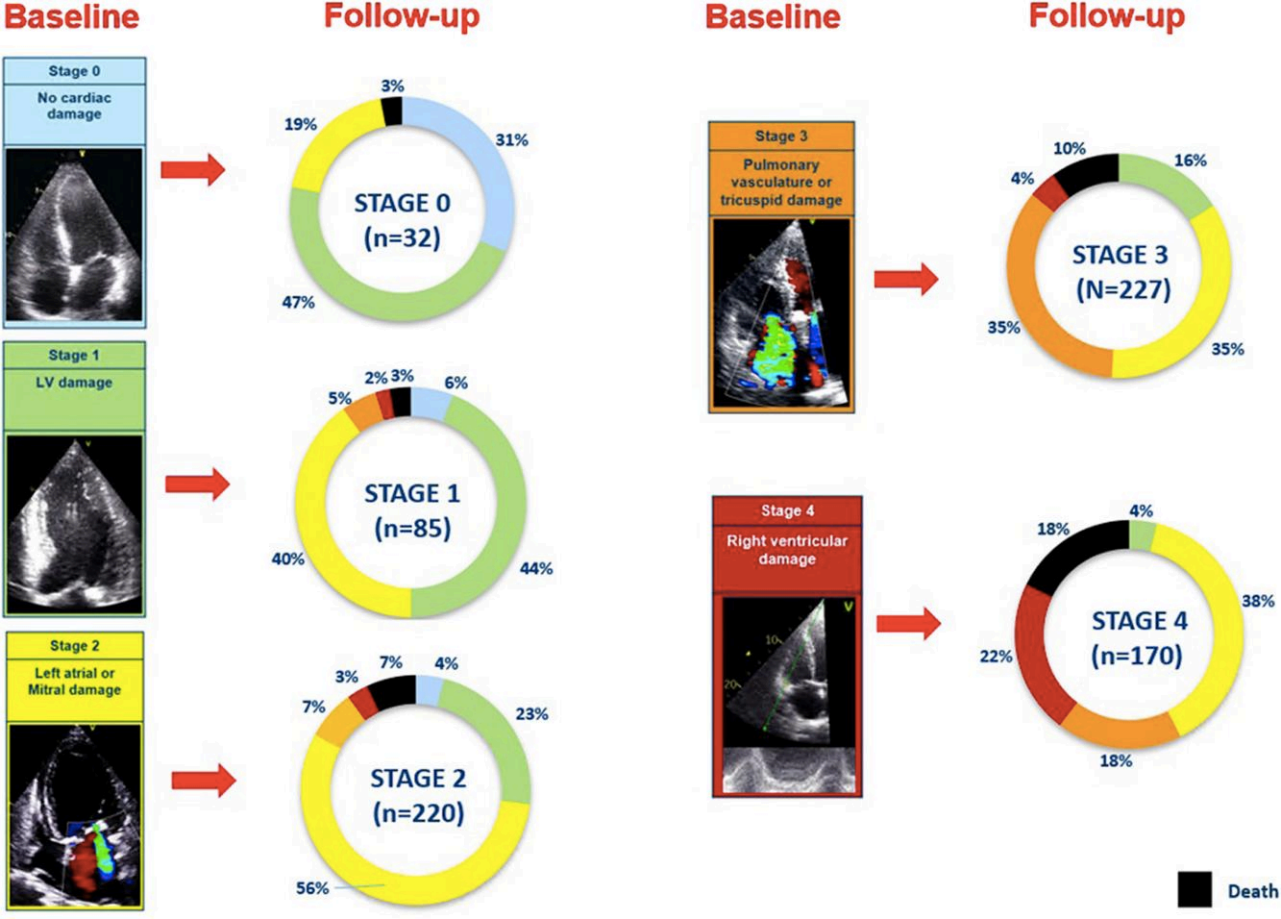
Evolution of baseline cardiac damage at 6-month follow-up per each stage. Each colour-coded rectangular box corresponds to baseline cardiac damage stages. Each pie chart shows the evolution of the baseline cardiac damage at 6-month follow-up after TAVI. Black colour denotes patients who died within 6 months after TAVI (*n* = 72). The other colours in the pie chart represent the cardiac damage stages as coded at baseline. LV, left ventricular; TAVI, transcatheter aortic valve implantation.

At 6-month follow-up, 72 patients died. Accordingly, 662 patients were available to compare echocardiographic parameters at baseline and 6 months after TAVI. Compared with the cardiac damage stage at baseline, 39.4% of the patients improved at least one stage at 6-month follow-up, while 38.1% remained in the same stage, and 12.7% showed worsening of at least one stage. *Figure 4*, Supplementary data online, *Figure S2* and Supplementary data online, *Table S2* displays the evolution of cardiac damage from baseline to 6 months after TAVI. After adjusting for EuroSCORE II, early vs. late TAVI (before compared with after 2015) and baseline stage of cardiac damage, the staging of cardiac damage at 6-month follow-up showed an independent association with all-cause mortality (HR per one-stage increase, 1.368; 95% CI, 1.002–1.867; *P* = 0.048; see *Table 3*, Model 2). To test whether the evolution of cardiac damage was independently associated with 2-year patient outcomes, an additional multivariable Cox regression analysis was performed testing the prognostic value of the 6-month changes in cardiac damage after TAVI adjusting it for the baseline cardiac damage staging and the EuroSCORE II. This analysis further confirmed the prognostic value of the change in cardiac damage stage between baseline (before TAVI) and 6 months after TAVI (*P* = 0.017; see *Table 3*, Model 3). Specifically, cardiac damage worsening (HR, 3.729; 95% CI, 1.494–9.305; *P* = 0.005) was associated with a higher risk of all-cause death compared to cardiac damage improvement 6 months after TAVI. Moreover, Supplementary data online, *Figure S3* shows the additional prognostic value of cardiac damage staged at 6-month follow-up (χ^2^ change = 4.317; *P* = 0.038) and cardiac damage evolution groups after TAVI (χ^2^ change = 8.627; *P* = 0.013) over the baseline assessment and EuroSCORE II.

**Table 3:** Independent associates of all-cause mortality at 2-year follow-up

Parameters	HR (95% Cl)	*P*-value
Model 1		
Euroscore II, per 1% increase	0.991 (0.948–1.037)	0.706
Late vs. early TAVI	0.503 (0.344–0.736)	**<0**.**001**
Staging at baseline (per 1 stage increase)	1.316 (1.097–1.577)	**0**.**003**
Model 2		
Euroscore II, per 1% increase	1.033 (0.981–1.089)	0.218
Late vs. early TAVI	0.574 (0.336–0.981)	**0**.**042**
Staging at baseline (per 1 stage increase)	0.948 (0.717–1.254)	0.710
Staging at 6 months (per 1 stage increase)	1.368 (1.002–1.867)	**0**.**048**
Model 3		
Euroscore II, per 1% increase	1.033 (0.981–1.089)	0.217
Late vs. early TAVI	0.581 (0.340–0.994)	**0**.**047**
Staging at baseline (per 1 stage increase)	1.391 (1.024–1.890)	**0**.**035**
Staging evolution at 6 months		
Improved	—	**0**.**017**
Stabilized	1.838 (0.993–3.401)	0.053
Worsened	3.729 (1.494–9.305)	**0**.**005**

Values are HR (95% CI). Bold values represent significant *P*-values (<0.05).

CI, confidence interval; EuroSCORE, European system for cardiac operative risk evaluation; HR, hazard ratio; TAVI, transcatheter aortic valve implantation.

## Discussion

The present study is the first to demonstrate the evolution and prognostic value of cardiac damage 6 months after TAVI in a real-world setting. Cardiac damage was highly prevalent before and after TAVI. Nevertheless, at 6-month follow-up, an improvement in cardiac damage was observed in almost 40% of the total population suggesting a high percentage of short-term reversibility of cardiac damage after TAVI. Cardiac damage staging at 6-month follow-up and its relative comparison with the assessment before TAVI had a significant independent association with 2-year all-cause mortality suggesting the importance of patient follow-up and longitudinal assessment to predict patient outcomes and improve risk stratification.

### Prevalence of extra-valvular cardiac damage in patients with severe AS

The cardiac damage staging system represents a holistic approach that takes into account all cardiac structures to quantify and stage the cardiac adverse remodelling and damage likely due to severe AS. The quantification of cardiac damage allows to risk stratify patients with severe AS and could be useful to identify the optimal timing for intervention before AS-related symptoms occur and irreversible cardiac damage takes place. In the present study depicting a real-world setting, cardiac damage before TAVI was nearly universal with only 4% of patients showing no cardiac damage and the remaining 96% with echocardiographic markers of LV (12%), LA and/or mitral valve (30%), pulmonary vasculature and/or tricuspid valve (31%), or RV damage (23%). Importantly, in accordance with previous studies,^20–22^ worsening cardiac damage stages before TAVI were associated with increased mortality even after aortic valve intervention. The ‘staging concept’ had been applied in various studies in patients with severe AS^7–9^ as well as other valvular heart diseases,^10,11^ showing a consistent prognostic value. Généreux *et al*.^12^ assessed the presence and evolution of cardiac damage before and 1 year after AVR in patients with severe AS undergoing surgical AVR or TAVI from 2 randomized clinical trials (PARTNER 2 and 3). The authors reported similar percentages of cardiac damage before AVR as noted in the current study, with a total of 94% of the patients presenting echocardiographic markers of cardiac damage before aortic valve intervention. In fact, in the study by Généreux *et al*.^12^ before AVR only 6% of the patients had no cardiac damage, 14% presented with LV damage, 51% with LA or mitral valve damage, 21% with pulmonary vasculature or tricuspid valve damage, and 7% with RV damage. The higher prevalence of RV damage in our study as compared to the study by Généreux *et al*.^12^ (23% vs. 7%, respectively) could be related to the highly selected populations in the PARTNER 2 and 3 trials, compared to the real-world setting presented in the current study. Using the same criteria to classify extra-valvular cardiac damage, Okuno *et al*.^20^ evaluated 1133 patients with severe AS undergoing TAVI and reported similar proportions of patients without cardiac damage (Stage 0, 3%), with LV damage (Stage 1, 10%), LA and/or mitral valve damage (Stage 2, 35%), pulmonary vasculature and/or tricuspid valve damage (Stage 3, 21%), or RV damage (Stage 4, 31%).^20^ The high prevalence of cardiac damage in patients undergoing AVR in the real-world setting may relate to the relatively late referral for treatment. Current guidelines recommend AVR in patients with severe AS only if they are symptomatic or in case of LV systolic dysfunction identified as a reduction in LV ejection fraction.^13^ However, symptoms and LV ejection fraction can be late markers of disease and irreversible cardiac remodelling may be present already in earlier stages. For instance, Vollema *et al*.^21^ using LV global longitudinal strain integrated in the staging system showed a prevalence of 91% of cardiac damage in a cohort of 616 asymptomatic patients with severe AS. Further confirming this hypothesis, Singh *et al*.^23^ showed the presence and progression of late gadolinium enhancement by cardiac magnetic resonance and therefore potentially irreversible cardiac damage, also in asymptomatic patients with moderate to severe AS.

### Prognostic implications of cardiac damage in AS

The cardiac damage staging system requires not only to focus on the aortic valve but also on the other cardiac structures to screen for cardiac damage associated with severe AS. This approach has a strong pathophysiological foundation and allows to follow-up patients rationally and to better understand their clinical status apart from symptoms which may be difficult to assess in elderly individuals who may suffer from several comorbidities that can make it difficult to understand whether the symptoms are related to AS or not.^24^ Similar to our study, Généreux *et al*.^12^ showed that both cardiac damage assessed before AVR and at 1-year follow-up after intervention were independent predictors of 2-year mortality. In the current study, including a large real-world cohort of patients undergoing TAVI, the presence of cardiac damage at 6-month follow-up was similarly associated with 2-year all-cause mortality even after adjusting for potential confounders. The current observations confirm the feasibility and value of risk stratification of the cardiac damage staging system in real-world clinical practice. Moreover, also the comparative assessment of cardiac damage between baseline and 6-month follow-up showed an independent association with prognosis demonstrating the importance of re-assessing cardiac damage after TAVI and comparing the findings with the pre-procedural status to improve risk stratification. Similarly to AS, also comorbidities such as atrial fibrillation, pulmonary hypertension, and coronary artery disease are associated with abnormal cardiac function,^25^ could independently contribute to cardiac damage and its evolution, can worsen prognosis and should also be taken into account for risk stratification in patients with severe AS undergoing TAVI and during follow-up.

### Reversibility of cardiac damage after TAVI

The current study showed a higher percentage of cardiac damage improvement after TAVI compared to the study by Généreux *et al*.^12^ (39% vs. 16% of patients). There could be several potential explanations for this observation. One reason could relate to the different patient populations: while Généreux *et al*.^12^ included patients from randomized controlled trials undergoing both TAVI and surgical AVR, the current study considered a real-world cohort including only patients who underwent TAVI. Moreover, in the current study, the presence of atrial fibrillation was not included in the staging model, since permanent atrial fibrillation is highly prevalent in patients undergoing TAVI, with very low likelihood of permanent conversion to sinus rhythm during follow-up.^12^

The degree and reversibility of cardiac damage in patients with severe AS are multifactorial. The LV adapts to the increased afterload posed by significant AS with compensatory LV hypertrophy. On the one hand, LV hypertrophy is beneficial since it allows the LV to develop higher pressures to maintain the stroke volume, cardiac output and systemic perfusion despite the presence of AS.^26^ On the other hand, LV hypertrophy can be accompanied by myocardial fibrosis that is not functional and can lead to the development of LV systolic and diastolic dysfunction as well as ventricular arrhythmias.^27^ Moreover, myocardial fibrosis represents a marker of LV irreversible damage that does not regress after AVR.^28^ Myocardial fibrosis can be detected with cardiac magnetic resonance imaging and is associated with heart failure as well as increased mortality in patients undergoing AVR.^29^ Puls *et al*.^30^ demonstrated that myocardial fibrosis assessed with endomyocardial biopsies in patients with AS correlates with the extent of pathological baseline cardiac remodelling, heart failure symptoms, and also adverse long-term cardiovascular prognosis after TAVI. Although echocardiography cannot detect myocardial fibrosis, it is useful to assess extra-valvular cardiac damage, which is associated with irreversible myocardial remodelling and damage. Therefore, it may be hypothesized that a more advanced cardiac damage stage may be related to a higher degree of myocardial fibrosis that may not regress after TAVI. This hypothesis, however, needs further validation.

### Clinical perspectives

This study highlights the importance of staging cardiac damage in patients with severe AS. The cardiac damage staging system can identify potential disease progression which may prompt the indication for TAVI before irreversible or less reversible cardiac damage has occurred and the potential benefit of TAVI may be blunted. The current findings suggest that TAVI should be considered before RV dysfunction (Stage 4) occurs. In addition, the presence of RV dysfunction can significantly deteriorate short-term outcomes. Moreover, the current findings highlight the importance of re-assessing extra-valvular damage at 6 months after TAVI. When RV dysfunction (Stage 4) persists, the risk of death remains high despite AVR. In patients without regression of cardiac damage after TAVI optimization of medical therapy including treatment of heart failure and concomitant comorbidities are important as they could play an even important role on residual risk and prognosis.

### Study limitations

The limitations of this retrospective single-centre study are inherent to the study design. Moreover, data on other imaging modalities that allow myocardial tissue characterization (e.g. cardiac magnetic resonance imaging) were not available and, therefore, the association between adverse and irreversible cardiac remodelling and the cardiac damage staging system based on echocardiography could not be investigated. A survival bias could have affected the analysis on the prognostic value of follow-up cardiac damage staging after TAVI.

## Conclusions

Although extra-valvular cardiac damage is highly prevalent in patients with severe AS undergoing TAVI, more than one-third of the patients show an improvement at 6 months after the procedure, which is associated with better outcomes. The echocardiography-based, extra-valvular cardiac damage staging assessed before and after TAVI may further improve risk stratification in patients undergoing TAVI.

## Supplementary Material

jeaf045_Supplementary_Data

## Data Availability

The data underlying this article will be shared on reasonable request to the corresponding author.
