# Supplementary material for: The reversibility of cardiac damage after transcatheter aortic valve implantation and short-term outcomes in a real-world setting
Source: Eur Heart J Cardiovasc Imaging. 2025 Feb 4;26(5):918–27. doi: 10.1093/ehjci/jeaf045 (PMC12042742; doi:10.1093/ehjci/jeaf045)
Supplement: jeaf045_Supplementary_Data [file jeaf045_supplementary_data.zip › Supplementary captions.docx]

**Supplementary Table 1S.** Uni- and multi-variable Cox regression analyses for 2-year all-cause mortality.

**Supplementary Table 2S.** Prevalence and evolution of cardiac damage stages at baseline and follow-up, with distribution across evolution groups by stage components.

**Supplementary Figure 1S. Kaplan-Meier survival analysis for all-cause mortality according to cardiac damage classified as proposed by Okuno et al. at baseline and follow-up.** While the prognosis of stage 3b at baseline was similar to stage 4, stage 3b at follow-up had a similar prognosis to stage 0, 1, 2 and 3a which was better than that of patients on stage 4.

**Supplementary Figure 2S. Alluvial plot showing the evolution of cardiac damage stages between baseline and 6-month follow-up**

Each color-coded rectangular box in the first bar corresponds to baseline cardiac damage stages while second bar corresponds to follow-up cardiac damage stages.

**Supplementary Figure 3S. Additional prognostic value of cardiac damage staging and staging variation after TAVI**

The figure shows the additional prognostic value of considering cardiac damage staging at 6-month follow-up after TAVI (model 2) and the variation of cardiac damage after TAVI (model 3) compared to a basal model including EuroSCORE II and cardiac damage staging at baseline.
