# Supplementary material for: The reversibility of cardiac damage after transcatheter aortic valve implantation and short-term outcomes in a real-world setting
Source: Eur Heart J Cardiovasc Imaging. 2025 Feb 4;26(5):918–27. doi: 10.1093/ehjci/jeaf045 (PMC12042742; doi:10.1093/ehjci/jeaf045)
Supplement: jeaf045_Supplementary_Data [file jeaf045_supplementary_data.zip › Supplementary tables.pdf]

**Supplementary Table 1S.** Uni- and multi-variable Cox regression analyses for 2-year all-cause mortality.

| | **Univariate analysis** | | **Multivariate analysis** | |
|---|---|---|---|---|
| | **HR (95% CI)** | **P-value** | **HR (95% CI)** | **P-value** |
| **Age, per 1 year increase** | 0.988 (0.965-1.011) | 0.305 | - | - |
| **Male gender** | 1.204 (0.838-1.729) | 0.315 | - | - |
| **Body mass index, per 1 kg/m$^2$ increase** | 0.982 (0.941-1.025) | 0.400 | - | - |
| **Coronary artery disease** | 1.478 (1.007-2.169) | **0.046** | 1.135 (0.742-1.736) | 0.560 |
| **Myocardial infarction** | 1.419 (0.954-2.109) | 0.084 | - | - |
| **Atrial fibrillation** | 1.547 (1.036-2.311) | **0.033** | 1.246 (0.792-1.958) | 0.342 |
| **Cardiac surgery** | 0.641 (0.388-1.057) | 0.081 | - | - |
| **Diabetes Mellitus** | 1.537 (1.061-2.226) | **0.023** | 1.445 (0.960-2.175) | 0.078 |
| **Hypertension** | 1.559 (0.973-2.497) | 0.065 | - | - |
| **Smoking** | 1.912 (1.297-2.818) | **0.001** | 1.331 (0.833-2.128) | 0.232 |
| **Peripheral artery disease** | 1.460 (1.005-2.123) | **0.047** | 1.158 (0.763-1.758) | 0.491 |
| **Chronic obstructive pulmonary disease** | 2.593 (1.768-3.805) | **<0.001** | 2.221 (1.457-3.386) | **<0.001** |
| **NYHA class III or IV** | 1.721 (1.158-2.559) | **0.007** | 1.542 (0.992-2.398) | 0.054 |
| **Diuretics** | 1.463 (1.001-2.139) | 0.050 | - | **-** |
| **EuroSCORE II, per 1% increase** | 1.017 (0.975-1.060) | 0.436 | - | **-** |
| **Hemoglobin, per 0.01 g/dL increase** | 0.834 (0.762-0.913) | **<0.001** | 0.857 (0.772-0.951) | **0.004** |
| **Creatinine, per 0.01 mg/dL increase** | 1.201 (1.063-1.357) | **0.003** | 1.172 (1.003-1.370) | **0.046** |
| **Late vs early TAVI** | 0.499 (0.342-0.729) | **<0.001** | 0.621 (0.402-0.959) | **0.032** |
| **Baseline staging, per 1 stage increase** | 1.313 (1.103-1.563) | **0.002** | 1.341 (1.098-1.637) | **0.004** |
| **Staging at 6-month (per 1 stage increase)*** | 1.408 (1.078-1.839) | **0.012** | - | **-** |

Bold values represent significant P-values (<0.05). *Landmark analysis was performed.

AS: Aortic stenosis; AVA: Aortic valve area; CI: Confidence interval; EuroSCORE: European system for cardiac operative risk evaluation; HR: Hazard ratio; NYHA: New York Heart Association. TAVI: Transcatheter aortic valve implantation.

**Supplementary Table 2S.** Prevalence and evolution of cardiac damage stages at baseline and follow-up, with distribution across evolution groups by stage components.

| | Baseline (n=734) | 6 months after TAVI (n=662) * | P-value | Baseline staging components by evolution groups | | |
|---|---|---|---|---|---|---|
| | | | | Improved (at least 1 stage) 280/734 (39.4%) | Stabilized 289/734 (38.1%) | Worsened (at least 1 stage) 93/734 (12.7%) |
| **Stage 0** | **32 (4)** | **23 (4)** | 0.029 | **-** | **10/289 (3)** | **21/93 (23)** |
| **Stage 1** | **85 (12)** | **144 (22)** | **<0.001** | **5/280 (2)** | **37/289 (13)** | **40/93 (43)** |
| **Increased LV mass index'** | 497 (69) | 405 (66) | 0.885 | 190/275 (69) | 199/287 (69) | 50/92 (54) |
| **E/e′ >14** | 447 (64) | 489 (75) | **<0.001** | 185/268 (69) | 163/275 (59) | 45/88 (51) |
| **LV ejection fraction <50%** | 222 (30) | 122 (20) | **<0.001** | 100/279 (36) | 72/288 (25) | 18/93 (19) |
| **Stage 2** | **220 (30)** | **309 (47)** | **<0.001** | **57/280 (20)** | **124/289 (43)** | **23/93 (25)** |
| **LA volume index >34 mL/m$^2$** | 532 (73) | 434 (67) | **0.009** | 231/278 (83) | 215/287 (75) | 31/93 (33) |
| **Moderate/severe MR** | 155 (22) | 81 (12) | **<0.001** | 71/278 (26) | 50/289 (18) | 10/92 (11) |
| **Stage 3** | **227 (31)** | **130 (20)** | **<0.001** | **116/280 (41)** | **80/289 (28)** | **9/93 (10)** |
| **PASP ≥60 mm Hg** | 37 (5) | 20 (3) | 0.090 | 16/280 (6) | 14/289 (5) | 1/93 (1) |
| **Moderate/severe TR** | 323 (44) | 145 (22) | **<0.001** | 169/280 (60) | 107/290 (37) | 9/93 (10) |
| **Stage 4** | **170 (23)** | **56 (9)** | **<0.001** | **102/280 (36)** | **38/289 (13)** | **-** |
| **Moderate/severe RV dysfunction** | 170 (23) | 56 (9) | **<0.001** | 102/280 (36) | 38/289 (13) | - |

Bold P-values depict significant differences (<0.05) between stages of cardiac damage at baseline and follow-up and calculated by McNemar test.

*72 patients died at 6-month follow-up.

'Increased LV mass index refers >95 g/m$^2$ for women, >115 g/m$^2$ for men.

LA: Left atrium; LV: Left ventricle; MR: Mitral regurgitation; PASP: Pulmonary artery systolic pressure; RV: Right ventricle; TR: Tricuspid regurgitation; TAVI: Transcatheter aortic valve implantation.
