# Supplementary figures and images for: The reversibility of cardiac damage after transcatheter aortic valve implantation and short-term outcomes in a real-world setting
Source: Eur Heart J Cardiovasc Imaging. 2025 Feb 4;26(5):918–27. doi: 10.1093/ehjci/jeaf045 (PMC12042742; doi:10.1093/ehjci/jeaf045)

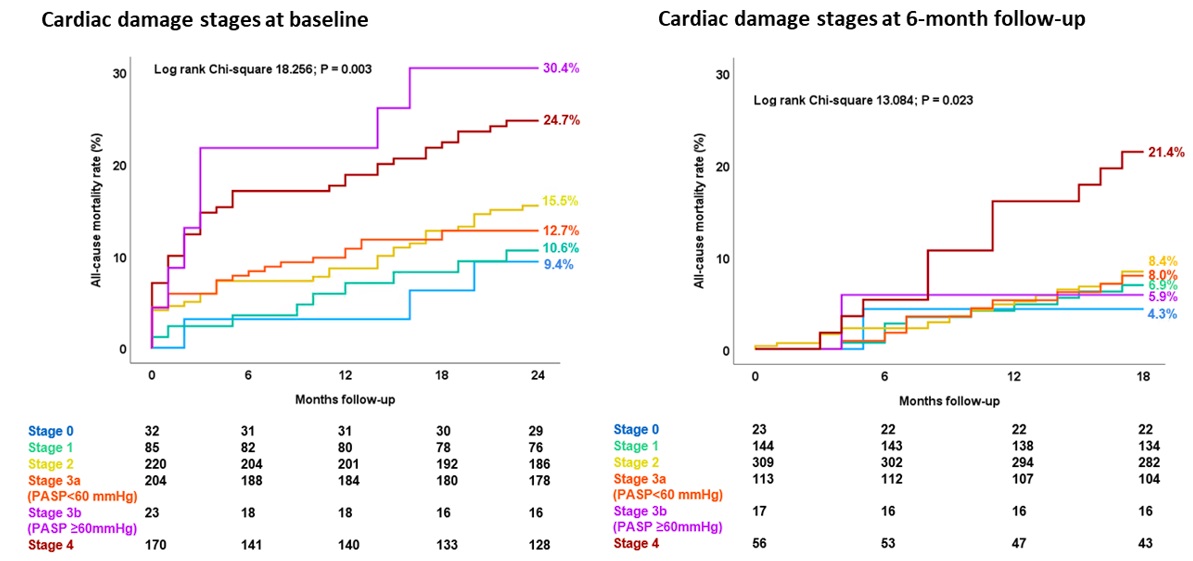

Supplement: jeaf045_Supplementary_Data [file jeaf045_supplementary_data.zip › Supp_Figure 1S..jpg]

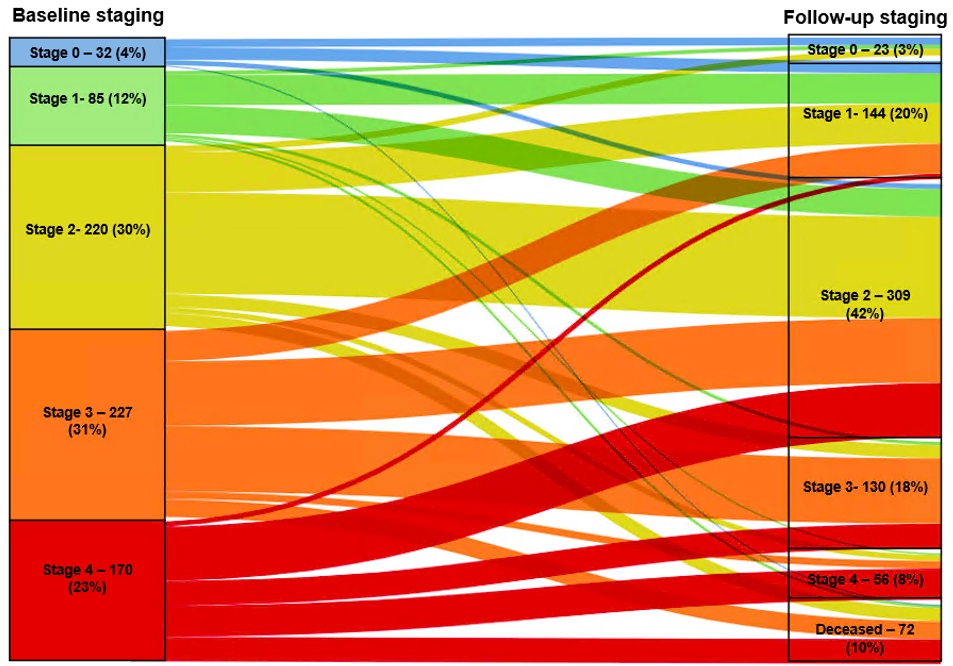

Supplement: jeaf045_Supplementary_Data [file jeaf045_supplementary_data.zip › Supp_Figure 2S.jpg]

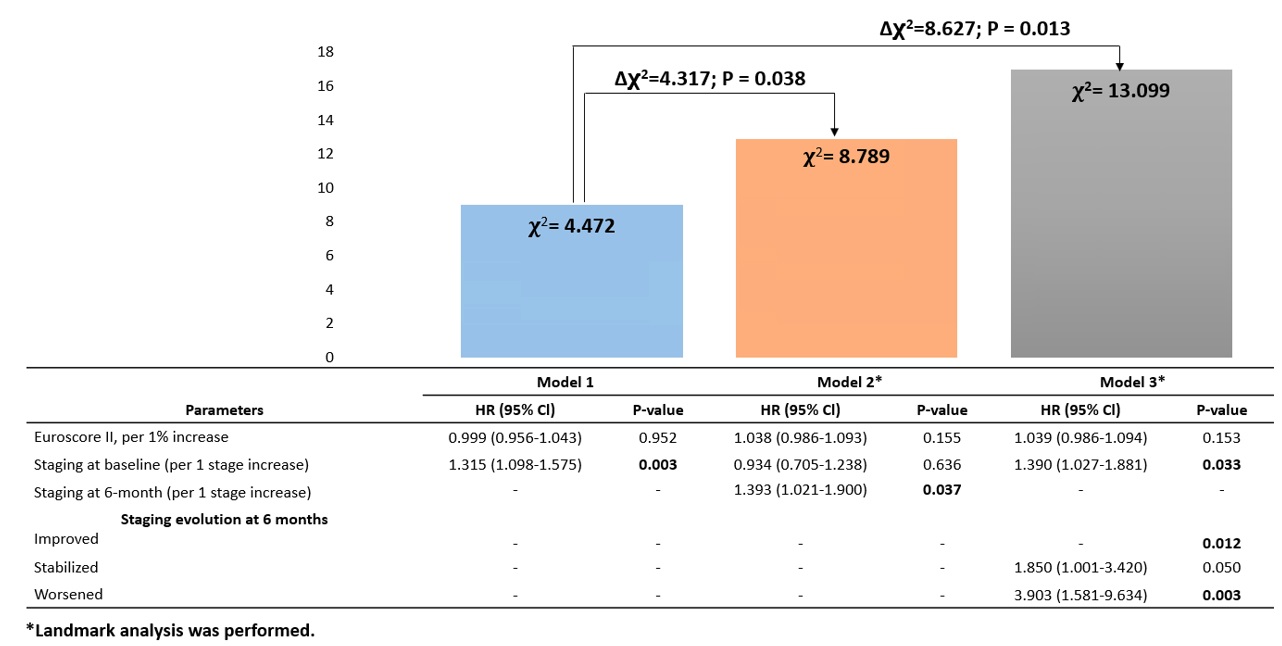

Supplement: jeaf045_Supplementary_Data [file jeaf045_supplementary_data.zip › Supp_Figure 3S.jpg]
